# Rethinking the representation of sound

**DOI:** 10.7554/eLife.82747

**Published:** 2022-09-07

**Authors:** Łukasz Bola

**Affiliations:** 1 Institute of Psychology, Polish Academy of Sciences Warsaw Poland

**Keywords:** early blindness, late blindness, brain plasticity, vision, audition, fMRI, Human

## Abstract

Blindness triggers a reorganization of the visual and auditory cortices in the brain.

**Related research article** Mattioni S, Rezk M, Battal C, Vadlamudi J, Collignon O. 2022. Impact of blindness onset on the representation of sound categories in occipital and temporal cortices. *eLife* **11**:e79370. doi: 10.7554/eLife.79370.

Humans are visual creatures, and making sense of what we see is a key task for many regions of the human brain. But what is the function of these regions in the brains of people who are blind? Now, in eLife, Stefania Mattioni, Olivier Collignon and colleagues at the University of Louvain report the results of experiments on blind and sighted participants which show that, in blindness, some regions that typically process visual signals are used to process sounds instead (Mattioni et al., 2022).

In sighted people, high-level visual areas in a region of the brain called the ventral occipitotemporal cortex sort incoming visual signals into different categories, with the distinctions between animate and inanimate, and between human and non-human, being important organizational principles (Konkle and Caramazza, 2013). High-level auditory areas in a nearby region of the brain, the superior temporal cortex, do the same for incoming sounds, and these two high-level areas are also known to communicate with each other (Adam and Noppeney, 2010).

Mattioni et al. used functional magnetic resonance imaging to study brain activity in participants as they listened to different types of sounds. In blind participants, the sounds activated the high-level visual areas more strongly than they did in sighted participants: this result is consistent with previous studies (such as Wang et al., 2015). The researchers also studied the high-level auditory areas and found that the activation of these areas was stronger for sighted participants than for blind participants – the opposite of what was observed for the high-level visual areas.

To explore further Mattioni et al. investigated how the participants responded to different types of sounds. They used four different sound categories: humans, animals, manipulable objects and big objects/places. Moreover, each category had two sub-categories: a human sound, for example, could be a vocalization or a facial-expression sound (such as laughing), and an animal sound could be made by a bird or by a mammal. The analysis involved using a special algorithm called a “classifier” that tried to distinguish between the different sounds based on how they activated the high-level visual and auditory areas.

The researchers found that the activation of the visual area contained more information about the sound category in blind participants than in sighted participants, whereas the activation of the auditory area was more informative in sighted participants than in blind participants. This confirms that blindness results in changes to both the ventral occipitotemporal cortex (which contains the high-level visual areas) and the superior temporal cortex (which contains the high-level auditory areas).

Mattioni et al. also wanted to find out if there were any organizing principles that helped to explain how the brains of blind and sighted participants responded to the different categories of sounds. They found that a model that divides sounds into human and non-human sounds best described the activations observed in both the auditory areas (although the model accuracy in these areas was diminished compared with sighted participants) and the visual areas in blind participants. In sighted participants, by contrast, this “human/non-human” model accurately described activations observed in the auditory areas but not in the visual areas. These results show that the reorganization of visual and auditory cortices in blindness might be complementary: in blind people, the distinction between human and non-human sounds is less precise in the auditory areas, but, at the same time, can be found in some visual areas (see Figure 1).

**Figure 1.**
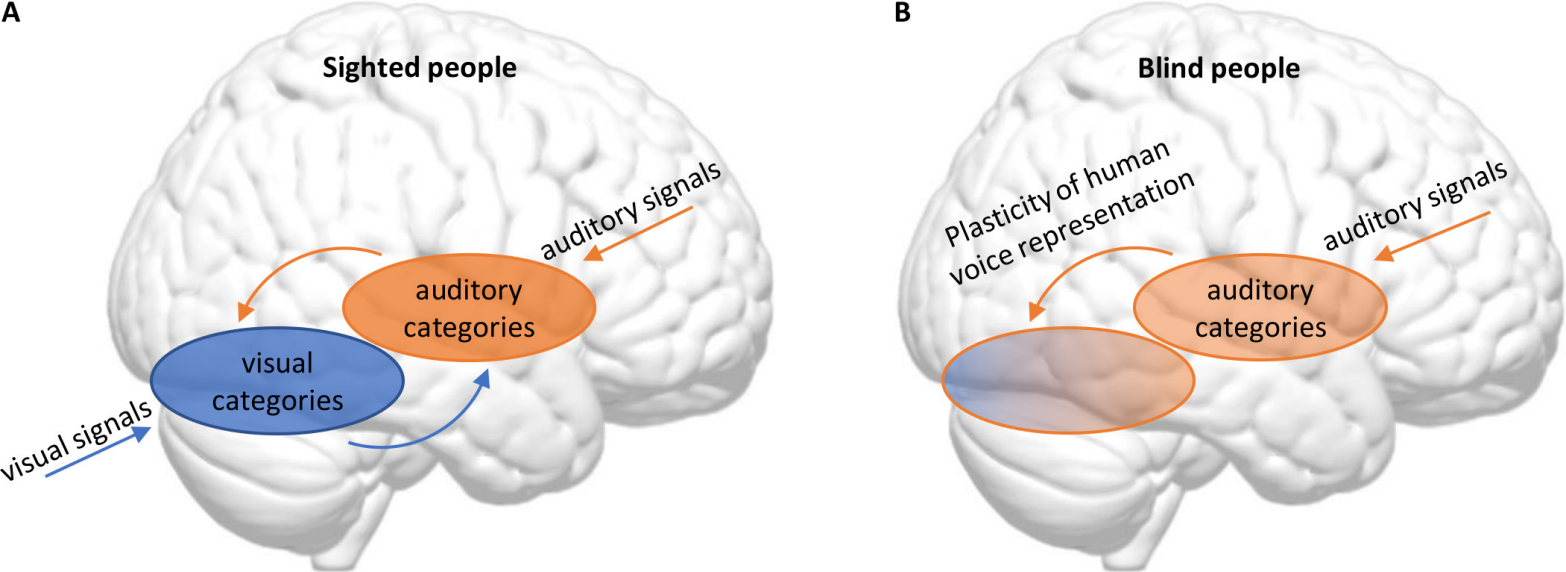
Blindness triggers a reorganization in the visual and auditory cortices in the brain. (**A**) In sighted people, high-level visual areas in the ventral occipitotemporal cortex (VOTC) sort incoming visual signals into categories (blue), and high-level auditory areas in the superior temporal cortex do the same with incoming sounds (orange). The high-level visual and auditory areas also communicate with each other. (**B**) Mattioni et al. found that, in blind participants, the high-level auditory areas (which are responsible for most of the processing of incoming sounds in sighted people) were less involved in sorting sounds into human and non-human sounds than in sighted participants. At the same time, parts of the VOTC that are primarily associated with visual processing in sighted people had become involved in sorting incoming sounds into these two categories.

Several studies have already hinted that the representation of sound in both the visual cortex and the auditory cortex undergoes a reorganization in blind people (Dormal et al., 2016; Jiang et al., 2016; van den Hurk et al., 2017; Vetter et al., 2020; Battal et al., 2022). Now Mattioni et al. have shown for the first time that these two processes might be linked and driven by a specific aspect of the sound (namely if it is produced by a human or not). Importantly, the researchers obtained very similar results in early-blind participants (who had been blind from an early age and reported having no visual memories) and late-blind participants (who did have visual memories). This shows that significant visual experience in the past does not preclude this type of reorganization, so the processes responsible for this reorganization are likely to rely on neural architecture that typically develops in sighted people.

As mentioned previously, when the high-level visual areas in the ventral occipitotemporal cortex are sorting incoming visual signals, the distinctions between animate/inanimate and human/non-human are important. Previous studies suggest that these high-level visual areas also contain some information about the category of incoming sounds in both blind and sighted people (van den Hurk et al., 2017; Mattioni et al., 2020). Now, Mattioni et al. have shown that some parts of the ventral occipitotemporal cortices are involved in sorting incoming sounds into categories (namely, human and non-human sounds) in blind participants only.

This calls for a detailed investigation of what is stable and what is plastic in the organization of this region. Could it be that some areas in this region – for example, those representing animate entities – are so dominated by visual signals that the auditory information in them only becomes apparent when vision is absent (Bi et al., 2016; Bola et al., 2022)? That would reconcile the hypothesis about the typical neural architecture supporting the reorganization processes studied by Mattioni et al. with the fact that, in sighted participants, no auditory effects were detected in the visual areas studied.

Other interesting questions spring to mind. What are the pathways that support joint reorganization of the visual and auditory cortices in blind people? Can observed neural changes explain better voice recognition abilities in blind people (Bull et al., 1983)? And can similar mechanisms guide reorganization in other visual regions (as suggested by Battal et al., 2022)? Whatever the ultimate answers to these questions might be, the work of Mattioni et al. illustrates an important point: understanding reorganization of the blind brain might require looking beyond the visual cortex.
